# Chloroquine-resistant *Plasmodium vivax *malaria in Serbo town, Jimma zone, south-west Ethiopia

**DOI:** 10.1186/1475-2875-8-177

**Published:** 2009-07-30

**Authors:** Tsige Ketema, Ketema Bacha, Tarekegn Birhanu, Beyene Petros

**Affiliations:** 1 Department of Biology, Addis Ababa University, P.O. Box 1176, Addis Ababa, Ethiopia; 2 Department of Biology, Jimma University, P.O. Box 378, Jimma, Ethiopia; 3 Department of Chemistry, Jimma University, P.O. Box 378, Jimma, Ethiopia; 4 Department of Chemistry, Addis Ababa University, P.O. Box 1176, Addis Ababa, Ethiopia

## Abstract

**Background:**

Ethiopia has the highest proportion of vivax malaria, approximately 40% of all malaria infections, in contrast to African countries. Chloroquine (CQ) is the drug of choice for the treatment of *Plasmodium vivax *infection in the country, although CQ resistant *P. vivax *(CRPv) has started to challenge the efficacy of the drug. The present study was conducted to assess the current status of CRPv at Serbo, Jimma zone, south-west Ethiopia.

**Methods:**

A 28-day *in vivo *therapeutic efficacy test was conducted from October 2007 to January 2008. Recurrence of parasitaemia and the clinical condition of patients were assessed on each visit during the follow-up. The levels of haemoglobin (Hb) in the study participants were determined. The patients' blood drug levels were measured using HPLC. Data was analysed using SPSS for windows version 10.0. HPLC data was computed using Chem Station for LC 3D systems software.

**Results:**

Of the total 84 patients included in the study, 78 completed their 28-day follow-up, six of whom being excluded for different reasons. In three children (aged 7, 12 and 13 years), parasitaemia reappeared within the 28-days follow-up in spite of adequate absorption of the drug and absence of malaria symptom. In addition, on the day of recurrence of parasitaemia the levels of chloroquine-desethylchloroquine (CQ-DCQ) were above the minimum effective concentration (≥100 ηg/ml) in all the three cases, showing that treatment failure could not be attributed to low level of drug in the patients blood.

**Conclusion:**

Reappearance of the parasite within the 28 days of follow-up is due to parasite resistance to CQ. The 3.6% (95% CI = -0.038 - 0.0758) prevalence of CRPv malaria in the study area signals the need for launching monitory activities for CQ resistant *P. vivax*. Moreover, as former report from the same country, Debrezeit, also showed the occurrence of CRPv, survey on CRPv malaria should be made in *P. vivax *endemic areas in order to estimate the level of burden across the country.

## Background

In Ethiopia, malaria is seasonal and unstable, causing frequent epidemics [1]. It usually occurs at altitudes < 2,000 meter above sea level. Occasionally, transmission of malaria occurs in areas previously free of malaria, including areas >2,000 meter above sea level, where the microclimate and weather conditions are not normally favourable for malaria. Recent report has indicated that epidemics have expanded to areas up to 2,400 meter above sea level [2].

*Plasmodium falciparum *and *P. vivax *are the two species commonly known to cause malaria in Ethiopia accounting for 60% and 40% proportion, respectively [3]. Resistance to anti-malarial drug in *P. falciparum *is well known since its first report on chloroquine (CQ) in 1998. The resistance to chloroquine (CQ) necessitated a change to sulphadoxine-pyrimethamine (SP) as first-line anti-malarial in Ethiopia. However, recent data has showed high mean SP treatment failure with 72% rate in some areas [4]. Consequently, making another change to artemether-lumefantrine (AL) was suggested in 2004. Until now there has been no report on the parasites resistance to the last drug [5].

Currently, CQ has been used as first-line drug for treatment of *P. vivax *infection throughout the country. However, chloroquine-resistant *P. vivax *(CRPv) has been emerging in different parts of the world. The first report of *P. vivax *resistant to chloroquine was from Papua New Guinea in 1989 [6], 30 years after resistance to this drug was first detected in *P. falciparum*. In the year that followed, isolated incidents of CRPv infections were reported from Irian Jaya, Indonesia, in 1991 [7], West Kalimantan, Indonesia, in 1998 [8], India in 2000 [9], Vietnam in 2000[10], Colombia in 2001 [11], Papua Indonesia in 2003 [12], Peru in 2003 [13], Brazil in 2007[14], and Dawei, southern Myanmar in 2008[15].

Since *P. vivax *malaria is rare in most part of the African continent, reports associated with *P. vivax *drug resistance, particularly resistance to CQ is scanty. To the authors' knowledge, there are only three reports: one from Madagascar [16] and the other two from Ethiopia [17,18]. The first report on CQ-resistant *P. vivax *in Ethiopia was from Debrezeit [17]. According to this report, of 225 patients who were followed for seven days, 2% failed to respond to treatment. A very detailed study was also conducted in the same site after 11 years [18]. In the later study, among 87 subjects involved in the study, parasitaemia re-appeared in four patients (5%) on day 28 during follow-up. The level of CQ plus its major metabolite in these subjects were above minimum effective concentration (MEC), which could confirm presence of drug resistant *P. vivax *parasite in the study area [19]. Therefore, the present study was conducted to assess the current status of CRPv at Serbo, Jimma zone, southwest Ethiopia. The result of this study showed that, in three children (aged 7, 12 and 13 years), parasitaemia re-appeared within 28-days follow-up in spite of adequate drug absorption and drug level above minimum effective concentration.

## Methods

### Study site

**Figure 1:**
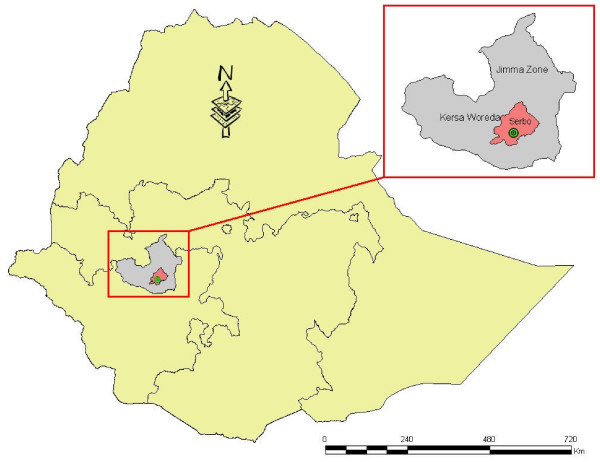
**Map of the study site**.

The study was conducted in Serbo Health Center found in Serbo town, Kersa woreda, Jimma zone, 345 km southwest of Addis Ababa from October, 2007 to June, 2008 (Figure 1). The study area is located between latitudes 7°35' – 8°00'N, and between longitudes 36°46'-37°14' E, at altitudes between 1,740 – 2,660 m above sea level [20]. It has an estimated total population of 329,629 of which 85% are dependent on agriculture, mainly growing coffee, chat, maize and fruits [20]. The major health problem in the woreda is malaria (77%). As the six consecutive years data (2002–2007) from Serbo Health Center showed, the number of malaria cases ranged between 3,925 and 22,938, with the peak being during 2004/5. *Plasmodium falciparum *(60%) has been more prevalent than *P. vivax *(40%), although the overall trend was similar for the two species. The prevalence seems decreasing although the number of cases per year is still high. The period from September to January is considered as the peak season of malaria transmission in the area. *Anopheles arabiensis *is a malaria vector found in Jimma zone [21].

### Study participants

Suspected patients seeking medication in Serbo Health Center were examined for the presence of *P. vivax *parasite monoinfection, on thick and thin film preparations, with a threshold of 250 parasites/μl or above. Among the screened patients, those who fulfilled inclusion criteria set by WHO [22] were recruited for the 28-days *in vivo *study.

The sample size was calculated based on the expected proportion of *P. vivax *treatment failures with CQ in the study population. Assuming a maximum of 5% treatment failures in a population of infinite size, a precision of +5%, and a 5% significance level, and expecting loss to follow-up rate of 15% over a 28-day study, 84 study participants were included [23].

### Treatment and follow-up

#### *In-vivo *test

A 28 days *in-vivo *drug efficacy testing was done according to the methods recommended by WHO [22]. An *in-vivo *test included treatment of symptomatic and parasitaemic individuals with a known dose of chloroquine, (25 mg base/kg), administered for three consecutive days (10 mg/kg on days 0 and 1, and 5 mg/kg on day 2) and the successive monitoring of the parasitological and/or clinical response overtime (for 28 days). All drug doses were administered under supervision of professionals and physiological complains were recorded at the time of each visit. Subjects were checked for vomiting for 30 minutes after ingestion of the drug; those who vomited the first dose were re-treated with an identical dose provided that the subject vomited the entire ingested drug. Subjects who vomited twice were dropped from the study. The study participants were advised not to take other drugs, except that patients with axillary temperature ≥ 37.5°C were treated with paracetamol.

Patients were asked to return for follow-up on days 1, 2, 3, 7, 14, 21, 28, for clinical examination including temperature recording, and on any occasion of malaria like illness [24]. Thick and thin blood smears were prepared at the time of all follow-up visits except day1. About 100 μl of blood sample was collected using EDTA heparinized capillary tube from lancet pricked finger on filter paper on days 0, 2 and any day when patients had recurrent parasitaemia for measurement of whole blood CQ and DCQ concentrations.

Patients who did not come for follow-up were traced to their homes. Those who failed to respond to CQ were re-treated with quinine sulphate (8 mg base/Kg) plus tetracycline (250 mg), for children older than eight years, and quinine sulphate plus clindamycine (300 mg) for children less than eight years of age three times daily for five days [13]. All re-treatments were done under supervision. The patients were followed for five days for any complains and released after the blood films of the cases were checked for parasite clearance.

### Laboratory procedure

#### Drug quality analysis

The quality of drug used in the study (chloroquine phosphate 250 mg, batch number 1482, Adigrat Pharmaceutical Factory) was tested for weight variation, disintegration, dissolution, identification and chloroquine hydrochloride injection assay, using the standard procedures recommended by British pharmacopoeia [25,26] at the National Laboratory of Drug Administration and Control Authority of Ethiopia. The batch of drug used for *in vivo *efficacy test was confirmed to fulfil all international specifications set for each mentioned tests.

#### Parasite identification

Parasites were identified by microscopic observation of the parasite's morphology using duplicate thick and thin blood smears taken at enrolment (Day 0). Subsequently, blood slides were done at subsequent visits (scheduled and non-scheduled). After fixing the thin film in methanol, both smears were stained with Giemsa (3%, pH 7.2) for 45 min and the thick films were examined under oil-immersion for malaria parasites. *P. vivax *asexual stages were counted against 200 white blood cells (WBC) or 500 WBC, if the number of asexual parasites was below 10 per 200 WBC, assuming the median total WBC count of 8,000/μl for the study population. Parasite reduction ratio (PRR) on day of admission and day 2 (after 48 hours), was evaluated as suggested by White [27] using the formula τ = P_o_/P_2_formula (where P_o _is parasite count on day 0 and P_2 _parasite count on day 2). Moreover, gametocytes were counted per 1,000 WBC, based on a mean WBC count of 8000/μl. Haemoglobin measurement was made on day 0 and 28 during follow-up and recurrence of parasitaemia using Hemocue™ (haemoglobinometer, Angelholm, Sweden).

Two experienced laboratory technicians working in Serbo health center examined each blood smear. All *P. vivax*-positive slides on day of admission, 10% of negative slides picked randomly from slides prepared during follow-up and all slides on day of recurrence were re-examined by senior laboratory technician.

### Endpoints

Study participants were classified either as 'adequate clinical and parasitological responses', in the absence of parasite recurrence within 28 days of follow-up, or 'treatment failure' in case of recurrence of parasitaemia during follow-up (within 28-days). When failures (whatever the genotype, either same or different) occurred in the presence of blood chloroquine concentrations (CQ+ DCQ) above100 ηg/ml, and adequate drug absorption, they were considered as resistant to CQ.

### Chloroquine and desethylchloroquine (DCQ) levels in whole blood

Blood drug levels (CQ-DCQ) in patients with recurrent *P. vivax *were measured using HPLC (Agilent 1200 series, Agilent Technologies, Germany) following the method described by Bell *et al *[28] with minor modification whereby the venous blood was replaced by blood spotted on filter paper. The LC-system of the HPLC instrument used was ZORBAX Ecllpse XDB-C18 reversed-phase analytical column (4.6 × 150 mm ID, 5 μm), with flow rate of 1 ml/min using isocratic mobile phase containing De-ionized water, acetonitrile and triethylamine in percent ratio of 90%, 10% and 1%, respectively.

### Data analysis

Data collected from *in vivo *therapeutic efficacy test was double entered and analyzed using SPSS software (version 10.0, Chicago, IL, USA). Kaplan-Meier survival probability analysis was used to evaluate treatment out come of study participants during follow-up period. Accordingly, data of patients excluded from the study for different reasons, including loss to follow-up, re-infection with *P. falciparum *and vomiting, were included in the analysis considering them as non-treatment failure cases. Relationship between parasite load and age of study participants on day of admission was compared using Pearson's correlation. Haemoglobin recovery of patients with adequate clinical and parasitological responses was compared using Mann-Whitney U test. Proportion of anaemic cases on day of admission and 28 were evaluated using Chi-Square test. In non-normally distributed data (age), median was used to measure the central tendency. In all analysis, significance level was considered at 95% confidence interval. In addition, HPLC data were computed using Chem Station for LC 3D systems software.

### Ethical clearance

The study protocol was reviewed and approved by the Ethical Review Committee of Addis Ababa University, Department of Biology. Written informed consent was obtained from each patients or guardian parent, in cases when the study subjects were younger than 18 years.

## Results

### Characteristics of the study population

During the study period, a total of 2,165 patients were examined for malaria infection and 692 (32%) were positive for malaria. Of the positive cases, 26.3% (182/692) were with *P. vivax *mono-infection; 84 of them who met all inclusion criteria set by WHO [22] were enrolled in the study. Median age of the study population was 8 years (9 months to 45 years), and the majority (60.7%, n = 51) of the cases were males (Table 1). Moreover, large number of the study population (75%, n = 63) were children <15 years. Of the total 84 cases included in the study, six cases were excluded during the 28 days follow-up for different reasons: three of them were re-infected with *P. falciparum *on day 14, 21, and 28, respectively were re-treated with artemether-lumefantrine (AL) or Coartem^®^; two cases were loss to follow-up on day 7 and one case re-vomited his second dose on day 1 (Table 2). Only 78 cases completed their 28 days follow-up.

**Table 1:** Demographic and clinical characteristics of the study participants, Serbo town, Jimma zone, Ethiopia

Characteristics	At day of admission (Day 0)
Median Age (years)	8 years (9 months – 45 years)
< 5	2 years (9 months – 4 years)
5–14	9 years (5 – 13 years)
15+	25 years (16 – 45 years)

Sex	
M	51 (60.7%)
F	33 (39.3%)

Mean Body Temp.	37.66°C

Febrile (Axillary Temp. ≥ 37°C)	46 (55%)

History of Fever	76 (90.5%)

Haemoglobin level	12.1 (7.5–16.8) ± 1.99 g/dl

Geometric mean parasite	2355.7/μl (95% CI = 408 – 4304)

Geometric mean of gametocyte	102.7/μl (95% CI = 66.3–139)

**Table 2:** Treatment out come of study participants during follow-up period in Serbo town, Jimma zone, southwest Ethiopia

Follow-up days	Population at risk	Excluded from the study	Treatment failure	Survival probability	95% confidence interval	
					
					Lower limit	Upper limit
Day 0	84	0	0	1	0.9455	1

Day 1	84	1	0	1	0.9455	1

Day 2	83	0	0	1	0.9455	1

Day 3	83	0	0	1	0.9455	1

Day 7	83	2	0	1	0.9455	1

Day 14	81	1	1	0.9876	0.9255	0.999

Day 21	79	1	1	0.9751	0.907	0.995

Day 28	77	1	1	0.9625	0.889	0.989

**Figure 2:**
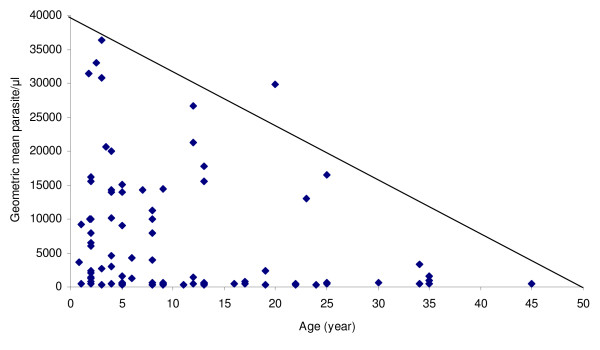
**Relationship between age group and parasite count at day of admission of study participants in Serbo town, Jimma zone, southwest Ethiopia**.

Geometric mean parasite count of the study participants were 2,355.7/μl (95% CI = 408 – 4304). Parasite counts were the highest (5,243.5 ± 10,831) in children <5 years as compared to other age groups. Among the study population, age and parasite density at day 0 were observed to have negative correlation (r = -0.276, significant at p = 0.01) (Figure 2).

### Treatment response

Since CQ is a fast-acting blood schizonticide drug, parasite clearance was achieved within 48 to 72 hours in 88% (n = 74) of study participants. The mean parasite reduction ratio (PRR) was 227.2/μl after 48 hour (on day 2). Complete parasite clearance was achieved on day 7 in all cases. Gametocytes were detected in 12% (n = 10) of the patients' blood films. In all cases, gametocyte continued to appear until day 2 and 3, but cleared on day 7 and did not re-appear thereafter until the end of the 28 days follow-up period.

Most of the study participants, 55% (n = 46) were febrile, had axillary temperature ≥ 37.5°C on day of admission, although the number shown declining to 34.6%, 29.5%, 10.3% on day 1, 2, and 3, respectively. About 8.3% (n = 7) of the total study participants were febrile on the remaining days (day 7 and 14) of follow-up. About 90.5% (n = 76) had history of fever for about 48 hours before admission. Except the 8.9% recorded on day 0 only less than 3% of the study participant had diarrhoea on day 1 and 2. Similar to the pattern observed for fever, the number of study participants who had history of vomiting was the highest on day 0 (37%, n = 31), but the number decreased down to 21.8% (day 1), 10.3% (day 2), 3.8% (day 3) and disappeared on day 7 in all cases.

Among patients treated with CQ at therapeutic dose under supervision, three of them revealed recurrent parasitaemia within the 28 days follow-up (on day 14, 25, and 28). In all patients with treatment failure, parasite count increased from day of admission to day of parasite recurrence (Table 3) without complains of malaria symptom except for the recurrence of parasitaemia. Only one patient (038) cleared parasites on day 7, while the other two cleared before 48 hours. In the former case, the parasite reduction ratio (PRR) after 48 hours was 2.375/μl.

**Table 3:** Parasite count, Haemoglobin level and CQ-DCQ level in the blood of *Plasmodium vivax *malaria patients with treatment failure at Serbo, Jimma zone, Ethiopia.

Patient's Code (day of Recurrence)	Parasite count/μl		Haemoglobin level (g/dl)		Level of CQ + DCQ (ηg/ml)		
	
	Day 0	Day of recurrence	Day 0	Day of recurrence	Day 0	Day 2	Day of recurrence
038 (28)	480	1920	11.6	11.6	ND	1391.2	145.99

054 (14)	480	960	14.2	14.2	10*	986.5	361.5

057 (25)	680	1040	10.7	12.5	ND	713.4	291.7

Note: ND = not detected; * Only CQ

### Haemoglobin level determination

**Figure 3:**
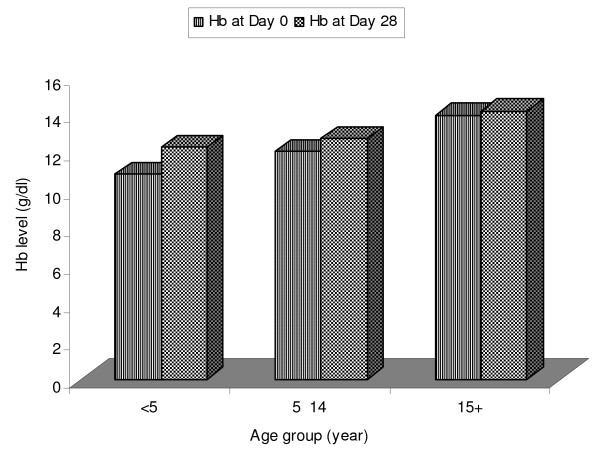
**Haemoglobin recoveries among study participants with adequate clinical and parasitological responses in Serbo town, Jimma zone, southwest Ethiopia**.

The Hb level on day of admission and day 28 were compared using Mann-Whitney U test and the result showed significant difference (p < 0.01) (Figure 3). Mean proportional recovery of haemoglobin among study participants with adequate clinical and parasitological responses was 0.11 g/dl. The proportion of anaemic cases on day of admission was not significantly different (χ^2 ^= 1.69, p = 0.193). However, on day 28, the number of anaemic cases differed significantly (χ^2 ^= 4.41, p = 0.035). But in the two patients with treatment failure, there were no difference in Hb level on day 0 and recurrence. In one of the patients with treatment failure the level of Hb was improved despite an increment in level of parasitaemia on day of recurrence (Table 3).

### CQ-DCQ level in patients with treatment failure

Results of the method validation test revealed that the total percent Coefficient of variation (%CV) of quality control samples were < 10% in all runs for each analyte (CQ and DCQ). Recovery of each analyte was 82.38% and 78.5%, respectively. Calibration curve of excellent linearity was obtained with correlation coefficient 0.995 and 0.998 for CQ and DCQ respectively. The peak area ratio of analyte (CQ and DCQ) in blood of patients with treatment failure against internal standard (QND) was considered to quantify the level of drug in their blood. CQ-DCQ levels in blood of patients with treatment failure showed that one of the patients was found exposed to CQ before the start of follow-up as small amount of CQ was detected in the patient's blood (Table 3). The three of them were found capable of absorbing the delivered CQ on day 2 as confirmed by detection of high concentration of CQ, 1030.37 ± 341 (713.4 -1391.2 ηg/ml) in their blood. In addition, blood of all the patients with treatment failure was found possessing CQ-DCQ level above the MEC on day of recurrence of parasitaemia (Table 3).

## Discussion

In the study conducted in Serbo Health Centre, three (n = 3) treatment failure was observed. Similar to the reports from Indonesia and Peru in which treatment failures were observed in children [8,13,29], the patients with treatment failure were also 7, 12, and 13 years. The increase in parasite density on day of parasite reappearance compared to enrolment day in cases with treatment failure was corresponding to the reports of Soto *et al*., and Baird *et al *[11,30] that showed parasitaemia on patients with treatment failure to increase at day of recurrence compared to that of the day of admission.

According to the protocol of WHO [22] for *in vivo *anti-malarial drug efficacy test, resistance is defined as the presentation of signs of severe malaria within the first two days after monitored treatment, the presence of parasitaemia and axillary temperature ≥ 37.5°C between day 3 and 28, presence of parasitaemia on any day between day 7 and 28, irrespective of clinical conditions. In this study all treatment failures were appeared on day between 7 and 28 and none had complained of malaria symptom. As anaemia is a major outcome of malaria illness and treatment with the appropriate drug will be expected to improve the patients' haemoglobin (Hb) level with time. However, in patients with treatment failure lack of Hb recovery on day of recurrence indirectly proves the presence of undetectable parasitaemia in their blood after treatment with CQ.

Since drug absorption problem of the patients could be a cause for the treatment failure, evaluation of the status of drug absorption of patients with treatment failure had shown that, the levels of CQ-DCQ was high on day 2 of CQ treatment. The high levels of drug in blood of the three patients with treatment failure showed lack of drug absorption problem. In addition in all cases drug level on day of recurrence was above MEC. Baird [19], stated that if the level of drug (CQ-DCQ) at day of recurrence is ≥ 100 ηg/ml, the reappeared parasite should be considered as resistant to CQ irrespective of its genotype (relapse, recrudesce or re-infection) and will be classified as chloroquine-resistant *P. vivax*. Therefore, based on the above criteria, the recurrent parasitaemia observed was due to resistance, as these parasites are present in the face of CQ level above the MEC.

In this study the three treatment failures [3.6% (95% CI = -0.038 - 0.0758)] observed was nearly similar to the study conducted at Debrezeit, Ethiopia (5%) [18]. However, the extent of resistance observed in this study was much lower as compared to the 41% (36/88) case from Iran Jaya, Indonesia [6], 84% from Papua, Indonesia [12], and 34% in Myanmar [15] where CRPv is common.

CQ is still drug of choice for treatment of *P. vivax *malaria in the study area. However, the emergence of CRPv malaria in the study area signals the need for regular monitoring activities of CQ resistant *P. vivax *in malaria endemic area of the country. The trend observed in Indonesia is good example to estimate the severity of the burden. A decade ago, the level of CRPv in Indonesia was similar to the current situation of Ethiopia. After 14 years, however, the level of CRPv increased to about 84% [12] in some area and enforced looking for other alternative therapy. Therefore, unless proper drug utilization is implemented and CRPv is monitored, the resistant strains could spread in the community within short period.

## Conclusion

This study confirmed the emergence of CRPv strains in Serbo town, Jimma zone, southwest Ethiopia. Moreover, as former report from the same country, Debrezeit, also showed the occurrence of CRPv, country wide survey on CRPv malaria should be made in *P. vivax *endemic areas for current estimate of the level of burden.

## Competing interests

The authors declare that they have no competing interests.

## Authors' contributions

TK was fully involved in all phases of the study, including data collection and monitoring both in the field and in laboratory during HPLC analysis, data analysis, interpretation, and write-up of the manuscript; BP designed the study project, supervised the study and critical revised the manuscript. KB was involved in statistical analysis of data and critical revision of the manuscript for publication; TB validated the HPLC method, analyzed and interpreted the HPLC data of the blood samples. All authors read and approved the final manuscript.
